# Determination of Effective Prophylactic Responses of Truncated Flagellin Protein as a Vaccine Candidate Against Uropathogenic *Escherichia coli*

**DOI:** 10.1155/ijm/9663212

**Published:** 2026-01-02

**Authors:** Aslam Dehvari, Zakaria Bameri, Mohammad Reza Asadi Karam, Ebrahim Kord, Mana Oloomi, Shahram Shahraki Zahedani, Mehri Habibi, Zabihollah Hashemzahi

**Affiliations:** ^1^ Infectious Diseases and Tropical Medicine Research Center, Research Institute of Cellular and Molecular Sciences in Infectious Diseases, Zahedan University of Medical Sciences, Zahedan, Iran, zaums.ac.ir; ^2^ Department of Molecular Biology, Pasteur Institute of Iran, Tehran, Iran, pasteur.ac.ir; ^3^ Biotechnology Department, Biotechnology Research Center, Pasteur Institute of Iran, Tehran, Iran, pasteur.ac.ir

**Keywords:** FimH.FliC, fusion protein, urinary tract infection, uropathogenic *Escherichia coli*

## Abstract

Uropathogenic *Escherichia coli* (UP*EC*) is a major cause of more than 80% of urinary tract infections (UTIs), a global health problem, and the second most common infectious disease. UTIs are responsible for approximately 40% of all nosocomial infections and 50% of all bacteremia. Since an approved vaccine against UTIs is not yet approved, evaluating different antigens, injection routes, and adjuvants is necessary to assess the ideal vaccines. In this study, we constructed a fusion protein composed of a truncated form of FimH antigen, the key virulence factor of UP*EC*, and the FliC antigen of *Salmonella typhimurium* as an adjuvant. After bioinformatics analysis, the fusion protein was cloned, expressed, and immunologically evaluated in mice. The bladder challenge assay was also used to examine the level of protection in the bladder and kidneys of the immunized mice. The levels of IgG and IgA antibodies in the serum and urine of mice vaccinated with the truncated FimH.FliC (tFimH.FliC) significantly increased compared to the FliC and PBS groups. The cytokine assay showed that tFimH.FliC fusion protein induced higher levels of IFN‐*γ*, IL‐4, and IL‐17 than the FimH and PBS groups. Additionally, the results of the challenge assay showed a significant decrease in the colony count of bacteria in all of the groups compared to the control group. Our findings showed that the designed candidate can develop effective prophylactic responses against UTIs caused by UP*EC* strains and that truncated FimH, without an unwanted domain, is an ideal vaccine target.

## 1. Introduction

Although various microorganisms, such as viruses, fungi, and bacteria, can cause urinary tract infections (UTIs), bacteria are responsible for more than 95% of UTIs [1]. Uropathogenic *Escherichia coli* (UP*EC*) as a leading cause of complicated and uncomplicated UTIs is responsible for more than 80% of all UTIs and is among the principal agents of death in children, the elderly, and women of all ages. UTIs cause serious consequences, including recurrence of infection, pyelonephritis leading to sepsis, premature birth, drug resistance, and pseudomembranous colitis. Additionally, the estimated cost of treating these infections is about $3.5 billion annually in the United States. This infection is the second most common infectious disease and is an inflammatory response at the urothelium surface against bacterial infection [2–4].

The pathogenesis of UP*EC* begins through the binding of FimH at the tip of the Type 1 fimbria and other binding factors with the receptors on the surface of the membrane of urinary tract cells. This binding protects the bacteria against the flow of urine, antibodies, bactericidal molecules, and antibiotic activity [5]. In addition to the prominent role of FimH in the attachment and colonization of bacteria, it performs a notable role in creating the intracellular form of UP*EC*, cell invasion, constructing a resistant biofilm‐like structure, and also causes inflammation in the urinary tract. This interaction of FimH results in evoking innate immune response and producing proinflammatory cytokines such as interleukin‐8 (IL‐8) [6]. Notably, their strains differ from others that are present in the gastrointestinal tract habitually. They have attained better adaptation to colonization in the urinary tract and escape from the host immune system responses [7]. Type 1 fimbriae are encoded by the fim structural operon, and their receptors are uroplakin, the bladder cell surface glycoprotein, and other proteins containing mannoside in the host [8, 9].

In addition to the high presence of the *fimH* gene in samples and conserved structure and sequences of this antigen, according to previous studies, this also has been shown that FimH possesses other properties of an ideal vaccine candidate against UTIs, including high immunogenicity, high expression at the site of infection, bacterial presentation, and a key role in pathogenesis [10–15].

The low immunogenicity is one of the reasons for the failure of vaccines developed against UTIs. New vaccine production technology using recombinant proteins reveals that many proteins lose their potential immune response stimulation features in the purified form. Thus, the adjuvant′s application is vital to increase its immunogenicity. Adjuvants accelerate the immune responses against the applied antigen by enhancing it and prolonging the duration of the immune response. It is also known that most adjuvants perform their function by mimicking pathogen‐associated molecular patterns (PAMPs) such as lipopolysaccharide (LPS), FimH adhesin, and flagella in pathogens [16, 17]. Bacterial flagellin protein (FliC), as the main flagellar protein, is one of the PAMPs. Its antigenic role in motile bacteria has been discussed for many years to induce a wide range of immune responses in the host. Therefore, it has also been a suitable target for vaccine design due to its role in pathogenesis [18, 19]. One of the advantages of FliC‐based fusion proteins is the requirement for fewer amounts of antigen and FliC, because FliC delivers the fusion protein directly to antigen‐presenting cells (APCs) by reacting with toll like recepor‐5 (TLR‐5) [20]. In addition, FliC has a stability property and, when attached to the proteins, can construct highly stable proteins [21].

Despite many efforts to develop a vaccine against UTIs, there is still no effective vaccine to prevent or treat UTIs [22–24]. Due to the necessity of an ideal vaccine to prevent UTIs, this study is aimed at designing a novel vaccine candidate through bioinformatics analyses using truncated antigens FimH and FliC and evaluating its immunogenicity and protection efficacy in a mouse model.

## 2. Material and Methods

### 2.1. tFimH.FliC Fusion Protein Construction

#### 2.1.1. Construction of tFimH.FliC Fusion Gene and Bioinformatics Evaluation

**Figure 1:**
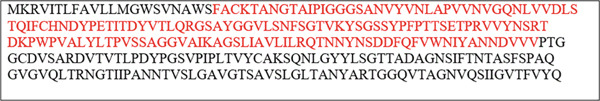
The amino acid sequence of the FimH. The positions of the amino acids used in the truncated FimH structure are highlighted in red.

We obtained the sequence of *fimH* and *fliC* genes from the national center for biotechnology information (NCBI) database (JX847135.1 and NC_016856, respectively). The polypeptide sequence of FimH is shown in Figure 1. Subsequently, the truncated sequence of the *fimH* gene (the N‐terminal domain), which is present in the outer part of bacteria with high immunogenicity [25], as well as the complete gene sequence of *fliC* from *Salmonella typhimurium*, was selected. These sequences were combined to form two models of the fusion protein including tFimH.FliC and FliC.tFimH. The I‐TASSER online server (http://wwwzhanglab.ccmb.med.umich.edu/I-TASSER) was used to model the fusion protein, which gives a three‐dimensional (3D) protein format and performs homology modeling. The I‐TASSER server predicted five hypothetical structures with a reliability coefficient (*C*‐score). Accordingly, the structure with the highest *C*‐score was chosen. Finally, the best‐determined structure of the fusion protein was determined by the YASARA server, and then energy minimization was performed.

The quality and efficacy of the predicted structures were evaluated using Expasy, ProSA, and Rampage servers. The ProSA server was used to determine the energy level of each model, according to its structure, and compared with other proteins that have been identified by X‐ray or NMR. The ProSA server also defines whether the model is in the range of known natural proteins or not, through the numerical coefficient *Z*‐score. Besides, the Expasy server specifies the physicochemical properties of proteins, such as molecular weight, isoelectric pH, stability, half‐life, hydrophilicity, and hydrophobicity (Table 1). The Rampage diagram was also employed to check whether the model contains unusual amino acids or not. The interaction of the fusion protein with TLR‐5 (as FliC receptor‐binding protein) was investigated by docking. Docking is aimed at investigating the position of the FliC in the protein fusion (first or end) for the best interaction with its receptor (TLR‐5). Hex Dock Version 8 software was used for docking.

**Table 1:** Chemophysical parameters of tFimH.FliC fusion protein.

**Parameters of tFimH.FliC**	
Number of a.a	651
Molecular weight	68,275.36
Isoelectric point (pI)	4.87
Formula	C_2946_H_4744_N_836_O_1015_S_5_
Number of atoms	9546
Estimated half‐life	30 h
Instability index^a^	27
Aliphatic index	85.01
GRAVY^b^	−0.286

^a^According to the Expasy ProtParam online tool criteria, these proteins are classified as stable.

^b^Grand average of hydropathicity (GRAVY), a smaller value shows that the protein is more hydrophilic.

#### 2.1.2. Production of tFimH.FliC Fusion Protein

The source clone of the *fimH* gene and *Salmonella typhimurium* ATCC 14028 standard strain from previous studies was used to construct tFimH.FliC fusion protein [21, 26]. BL21 (DE3) host containing the cloned *fimH* gene and *Salmonella typhimurium* ATCC1408 was cultured in 5 mL LB (Luria‐Bertani) medium (Merck), and after incubation for 18–24 h, the genome of *Salmonella typhimurium* and plasmid containing *fimH* gene were purified by the phenol–chloroform method and plasmid purification kit (Roche, Germany), respectively. The desired primers were designed to amplify *fimH* and *fliC* genes and introduce an *Nco*I site at the 5 ^′^‐terminus and a *Hind*III site at the 3 ^′^‐terminus of the genes (Table 2). Then, these genes were amplified based on our previous study using an optimized program and the *pfu* DNA polymerase (Fermentas, Lithuania) [26]. Moreover, sequencing was done for the amplified *fliC* gene and then compared with the NCBI‐registered gene sequence to confirm its accuracy. The amplified *fliC* gene was electrophoresed on a 1% agarose gel, and the PCR product was eluted from the gel by a Gel Purification Kit (GeneJET Gel Extraction Kit, Fermentas).

**Table 2:** List of primers in PCR and overlapped PCR to amplify the t*fimH.fliC* fusion gene and the *fliC* gene.

**Number**	**Primer name**	**Sequence (5** ^′^ **-3** ^′^ **)**
1	fliC for	5 ^′^‐CAT GCC ATG GCG ATG GCA CAA GTC ATT AAT‐3 ^′^
2	fliC rev	5 ^′^‐CCC AAG CTT ACG CAG TAA AGA GAG GAC‐3 ^′^
3	fimH for	5 ^′^‐GGT ATC GCA CCA CCA TTC GCC TGT AAA ACC‐3 ^′^
4	Fusion tfimH.fliC for	5 ^′^‐AAT GAT GTG GTG GTG ATG GCA CAA GTC ATT‐3 ^′^
5	Fusion tfimH.fliC rev	5 ^′^‐AAT GAC TTG TGC CAT CAC CAC CAC ATC ATT‐3 ^′^

For transformation, the pET28a plasmid was purified using a plasmid purification kit (Roche, Germany). Enzymatic cleavage of the extracted pET28a vector and PCR product (*fliC* gene) was performed with *Nco*I and *Hind*III enzymes. Ligation of the cleaved PCR product in the pET28a vector was performed using T4 DNA ligase (Fermentas). The ligation product was transformed into BL21 (DE3) competent cells. The transformed product was cultured on LB agar containing kanamycin for 18–24 h. Colony PCR, double digestion of the plasmid, and sequencing were performed to confirm the transformation. The *fliC* gene was sequenced and compared with the sequences in the GenBank, Expasy, BLAST (http://www.ncbi.nih.gov), and ClustalW (http://www.ebi.ac.uk/Tools/clustalw2). The fusion gene was constructed by attaching the N‐terminus of the *fliC* gene to the C‐terminus of the t*fimH* (truncated *fimH*) gene to produce the t*fimH.fliC* fusion gene using the overlapped PCR.

Protein expression was optimized by using different concentrations (0.1–1 mM) of IPTG inducer in the LB broth. SDS‐PAGE and western blotting techniques were applied to evaluate expressed proteins. Subsequently, protein purification was conducted using Ni‐NTA resin (QIAGEN). Due to the high level of endotoxin in the samples, different concentrations of Triton X‐114 were used to remove LPS [27].

The biological activity of the fusion protein was assessed in HT‐29 cells obtained from the National Cell Bank of Iran at the Pasteur Institute of Iran and incubated with the purified proteins. Subsequently, the IL‐8 level was assessed by the ELISA, according to the kit instructions (R&D Systems, United States).

### 2.2. Evaluation of the Immunogenicity of tFimH.FliC Fusion Protein in Mice

#### 2.2.1. Mice Immunization

Female BALB/c mice (6–8 weeks old) were prepared from the Pasteur Institute of Iran and kept under standard temperature and humidity conditions. The mice were divided into four groups, including tFimH.FliC, FimH alone, FliC alone, and control. Totally, 25 *μ*g of each protein was injected subcutaneously on Days 0, 14, and 28 into mice. The mice in the control group received PBS. Blood samples were taken from the injected mice at 2‐week intervals after the first, second, and third injections. Urine samples were also collected 2 weeks after the third injection. In all steps of animal studies, the guidelines of the European Communities Council (86/609/EEC) were used. The animal studies were performed according to the ethical safety guidelines of Zahedan University of Medical Sciences, Zahedan, Iran, under Ethical Number IR.ZAUMS.REC.1398.054.

#### 2.2.2. Evaluation of Vaccine Efficacy in Bladder Challenge

The challenge test was carried out according to a previous study [26]. *Escherichia coli* CFT073 strain was inoculated (10^8^ cfu/mL) transurethrally into the bladder through special catheters (BD INTRAMEDIC, Ref 427400, United States), 7 days after the third vaccine dose. A week later, the infected mice were sacrificed. The kidneys and bladders of the infected mice were homogenized in PBS. The suspension of homogenized organs was incubated on the LB agar medium. After 18–24 h incubation at 35°C–37°C, the colony count of bacteria was determined.

### 2.3. Assessment of Specific Immune Responses

#### 2.3.1. Antibody Detection

Serum and urine supernatants were used to assess total IgG, IgG2a, IgG1, and IgA isotype levels by ELISA, as described in the previous study [26]. Briefly, the FimH protein was coated onto 96‐well microtiter plates (2 *μ*g/mL). Serial dilutions of sera (1:50–1:5000) and urine samples (1:1–1:500) in blocking buffer were applied as primary antibodies and HRP‐conjugated antimouse IgA, total IgG, IgG1, and IgG2a (Sigma, United States) as secondary antibodies. The dilutions or concentrations of sera, urine, FimH antigen, and secondary antibodies were optimized separately in the current study.

#### 2.3.2. Cytokine Assay

Splenocytes were cultured in vitro with tFimH.FliC, FimH, and FliC proteins as described previously [26]. IL‐17, IFN‐*γ* (as indicators of Th1), and IL‐4 (as indicators of Th2) in the supernatants collected from the splenocytes were measured using Mouse DuoSet ELISA kits (R&D Systems Inc.). All steps of measuring were performed according to the manufacturer′s recommendations.

### 2.4. Statistical Analysis

One‐way ANOVA, two‐way ANOVA, Student *t*‐test, and Tukey HSD were used to statistically analyze the data. The GraphPad Prism was also used for bladder and kidney challenge analysis by comparing different groups through the Kruskal–Wallis (Dunn′s multiple comparison tests) test. In all statistical analyses, *p* < 0.05 was considered an indicator of significance.

## 3. Result

### 3.1. The tFimH.FliC Structure Was Selected for the Construction of the Fusion Protein

Based on models obtained by the I‐TASSER server, the tFimH.FliC structure with a coefficient of −3.15 (*C*‐score) had better quality than the FliC.tFimH structure. Also, the ProSA‐web determined that the 3D structure of this model had a higher convergence to the natural crystal structure (*Z* − score = −8.45). More information about the physicochemical properties of tFimH.FliC determined by the Expasy server is shown in Table 1. According to docking analysis, the interaction between the tFimH.FliC structure and TLR‐5 was better than FliC.tFimH.

### 3.2. The tFimH.FliC Fusion Protein Was Produced and Verified

Using the PCR technique, *fliC* and truncated *fimH* genes were amplified from *Salmonella typhimurium* (ATCC 14028) and related plasmid stored in the BL21 (DE3) strain, respectively, and fused. The 1488 and 495 bp bands corresponding to the complete *fliC* and truncated *fimH* genes were observed, respectively. Also, a 1980 bp band representing the t*fimH.fliC* gene was constructed by linking *fliC* and t*fimH* genes using overlapped PCR.

**Figure 2:**
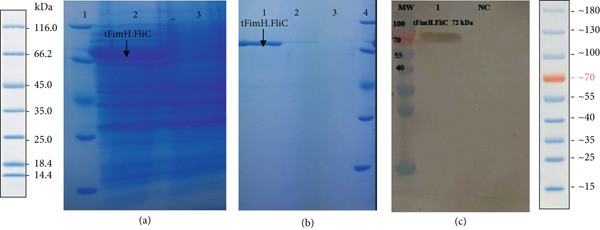
(a) SDS‐PAGE analysis of tFimH.FliC protein: Lane 1, ladder; Lane 2, expression of tFimH.FliC in *E. coli* BL21 (DH3); Lane 3, negative control (*E. coli* BL21) (DH3). (b) Protein purification: Lanes 1–2, tFimH.FliC fusion protein (72 kDa); Lane 3, negative control (*E. coli* BL21) (DE3); Lane 4, ladder. (c) Western blot analysis: MW, ladder; Lane 1, tFimH.FliC protein (72 kDa); NC, negative control (*E. coli* BL 21) (DE3).

**Figure 3:**
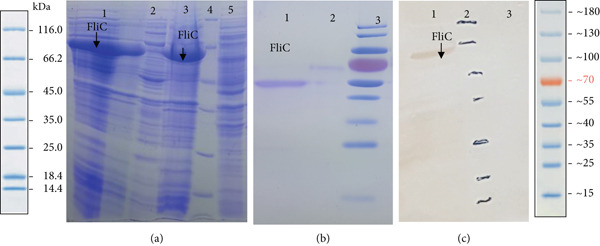
(a) SDS‐PAGE analysis of FliC protein: Lanes 1 and 3, expression of FliC in *E. coli* BL21 (DE3); Lanes 2 and 5, negative control (*E. coli* BL21); Lane 4, ladder. (b) Protein purification: Lane 1, FliC protein; Lane 2, negative control; Lane 3, ladder. (c) Western blot analysis: Lane 1, FliC protein (54 kDa); Lane 2, ladder; Lane 3, negative control (*E. coli* BL 21).

The t*fimH.fliC* fusion gene was cloned into the pET28a vector and expressed in the BL21 (DE3) strain. Colony PCR, enzyme digestion using *Nco*I and *Hind*III, and sequencing confirmed the cloning process. Furthermore, the gene expression was verified through the SDS‐PAGE and western blot, exhibiting specific protein bands 72 and 54 kDa for the tFimH.FliC and FliC proteins, respectively (Figures 2 and 3). It should be noted that the most desirable protein expression for the FliC and tFimH.FliC proteins resulted from incubation with 0.5 and 1 mM IPTG, respectively (Figures 2 and 3). The proteins were successfully purified by Ni‐NTA resin. The concentrations of tFimH.FliC and FliC were 150 and 200 *μ*g/mL, respectively. Additionally, the final LPS level after using the wash solution containing 0.1% Triton X‐114 was less than 0.1 EU/mL.

**Figure 4:**
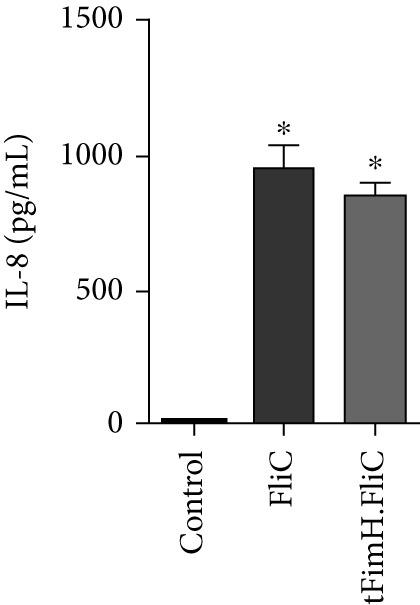
Production of IL‐8 in HT‐29 cell culture induced by FliC and tFimH.FliC proteins. The single asterisks indicate the statistical significance of FliC and tFimH.FliC compared to the control group (*p* < 0.001).

The biological activity of the purified proteins was assessed in the HT‐29 cell line. Production of IL‐8 in wells containing HT‐29 cell lines treated with tFimH.FliC and FliC proteins increased significantly compared to the control wells (*p* < 0.001) (Figure 4).

### 3.3. The tFimH.FliC Fusion Protein Induced Significant Antibody Responses

After the first subcutaneous injection of tFimH.FliC and FimH groups, the level of total IgG antibody increased slightly compared to the FliC and control groups, while following the second and third injections increased significantly (Figure 5a). The results after the third injection showed statistically more significant antibodies than the control and the FliC groups (*p* < 0.001) (Figure 5a). After the third vaccine dose, the IgG isotype (IgG1 and IgG2a) level in the mice groups that were immunized with tFimH.FliC and FimH alone was significantly higher than the FliC and PBS groups (*p* < 0.05) (Figure 5b). It is noteworthy that the level of IgG2a in tFimH.FliC group was significantly higher than the FimH group (*p* = 0.016) (Figure 5b).

Figure 5Evaluation of antibody responses. (a) Measurement of total IgG antibody in serum on Days 0, 14, and 28 after the first vaccine dose. The figure indicates the statistical significance of FimH and tFimH.FliC groups over FliC and PBS groups (*p* < 0.001) in the third serum. Single asterisks represent statistical significance of IgG in FimH and tFimH.FliC groups over the FliC and PBS in the second serum (*p* < 0.01). (b) Serum anti‐FimH IgG1 and IgG2a. This figure indicates statistical significance between FimH and tFimH.FliC groups over FliC and PBS groups (*p* < 0.05). Single asterisk represents the statistical significance of IgG2a in tFimH.FliC group over FimH alone (*p* < 0.01).

**Figure:**
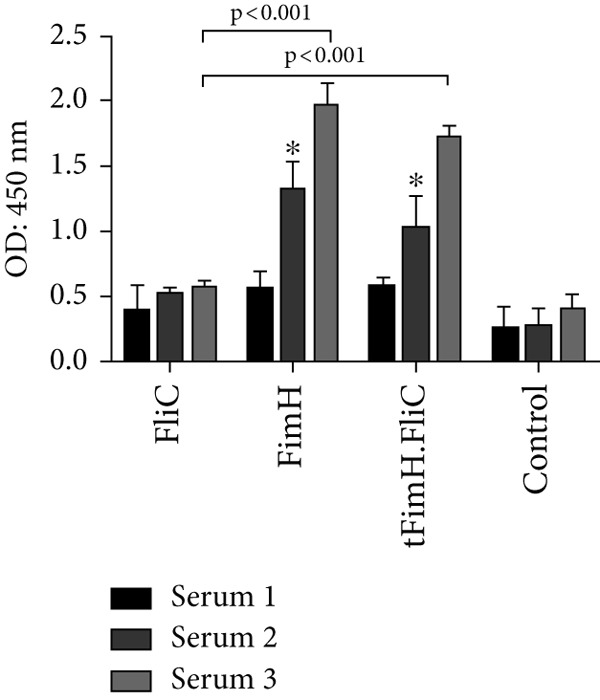
(a)

**Figure:**
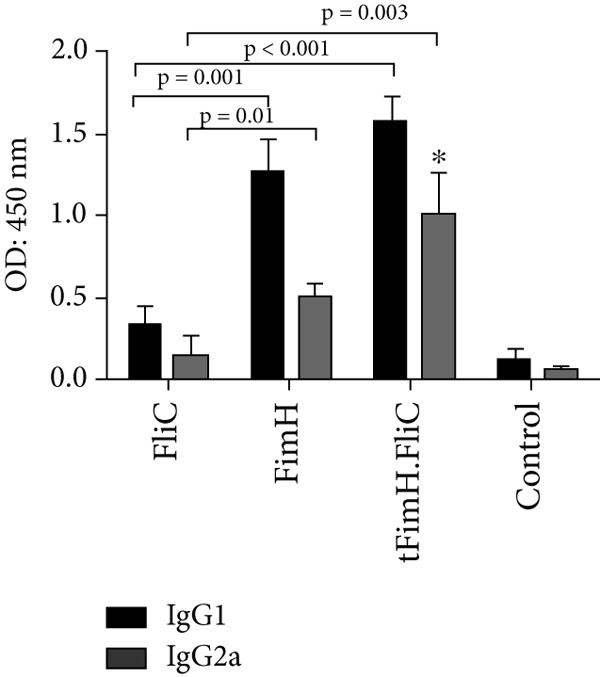
(b)

Evaluation of IgA levels in the collected serum and urine samples also revealed that mice immunized with tFimH.FliC or FimH alone produced a significant IgA level compared to the FliC and PBS groups (*p* < 0.001) (Figure 6). In addition, the tFimH.FliC protein induced more IgA than FimH, but this difference was not significant (*p* > 0.05) (Figure 6).

Figure 6Evaluation of serum and mucosal IgA. (a) Measurement of IgA antibody in serum. The results show statistical significance of tFimH.FliC and FimH over groups that received PBS and FliC (*p* < 0.001). (b) Measurement of IgA antibody in the urine. The results show statistical significance of tFimH.FliC and FimH over groups that received PBS and FliC protein (*p* < 0.001).

**Figure:**
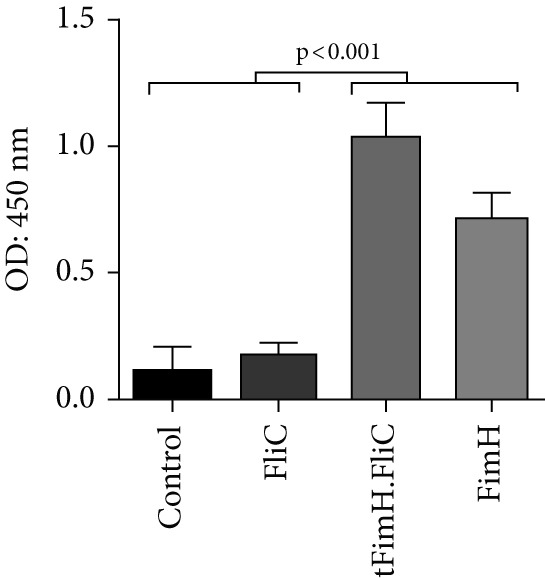
(a)

**Figure:**
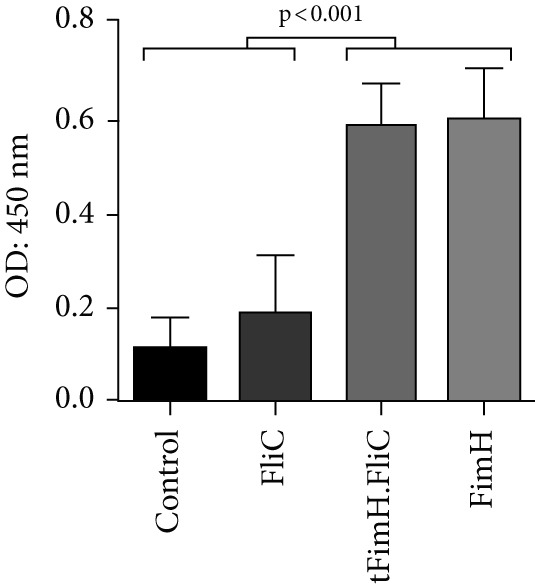
(b)

### 3.4. The tFimH.FliC Fusion Protein Induced Significant Cytokine Responses

To evaluate the relative cellular immune responses, the production of cytokines IFN‐*γ*, IL‐4, and IL‐17 in the splenocytes, after exposure to the specific antigens, was investigated by ELISA. The results showed that injection of tFimH.FliC, FliC, and FimH proteins resulted in significantly higher levels of IFN‐*γ*, IL‐4, and IL‐17 than the PBS (*p* < 0.05) (Figure 7). Also, the results showed the presence of FliC in tFimH.FliC fusion protein has caused a significant increase in IFN‐*γ*, IL‐17, and IL‐4 levels compared to the FimH (*p* < 0.05) (Figure 7). It was also found that mice received the tFimH.FliC protein, significantly increased the IL‐4 compared to the mice that received FliC alone (*p* = 0.001) (Figure 7c).

Figure 7Cytokine assessment in the splenocytes of mice. The collected splenocytes of mice were cultured in the presence of FimH, FliC, and tFimH.FliC proteins, and the cytokine levels were measured. (a) Measurement of IL‐17 level. Results present the statistical significance of tFimH.FliC over FimH (*p* = 0.01). (b) Measurement of IFN‐*γ* levels. The figure indicates that tFimH.FliC presents statistical significance over the FimH group (*p* = 0.003). (c) Measurement of IL‐4 levels. The results indicate the statistical significance of tFimH.FliC over the other groups (*p* < 0.05). The single asterisks indicate that IFN‐*γ*, IL‐17, and IL‐4 were significantly produced in tFimH.FliC, FliC, and FimH groups over the control group (*p* < 0.001).

**Figure:**
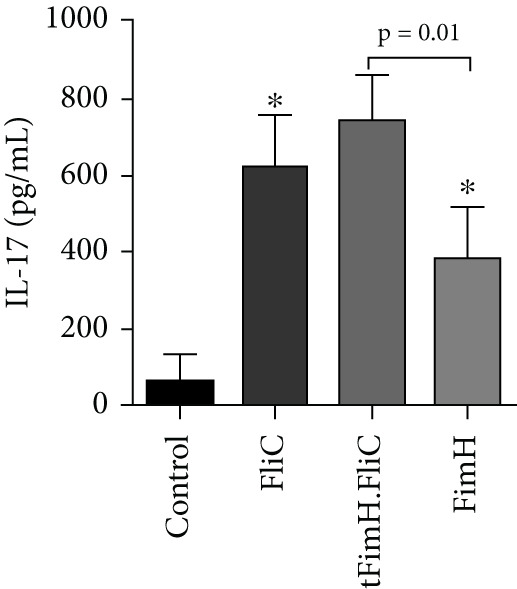
(a)

**Figure:**
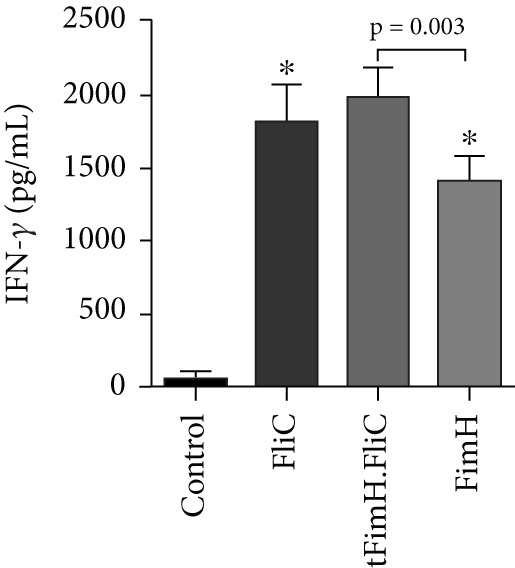
(b)

**Figure:**
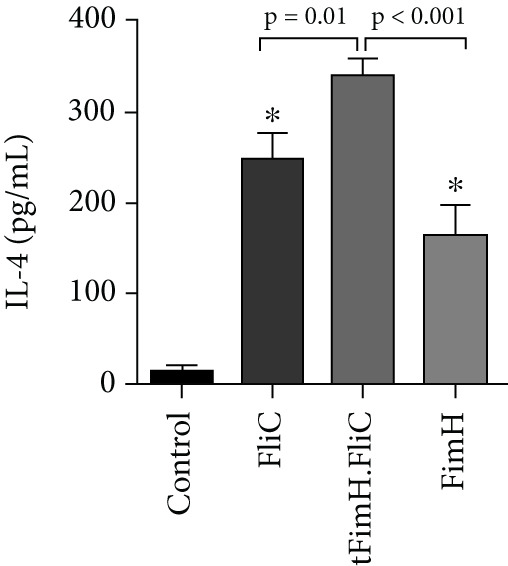
(c)

### 3.5. The tFimH.FliC Fusion Protein Provides Protection Against UP*EC* Colonization

To evaluate the efficiency of the immune responses provided in the vaccinated mice, a challenge assay was performed. The results of bacterial colony counting in the bladder challenge assay showed that all groups could significantly reduce UP*EC* colonization in the bladder and kidneys compared to the control group (*p* < 0.05) (Figure 8). Comparison of colony counts among the vaccinated groups showed that the tFimH.FliC group had the lowest colony count. Of course, it should be noted that the number of colonies in tFimH.FliC and also the FimH group was significantly lower than the FliC group (*p* < 0.05).

Figure 8Colony count of UP*EC* in the infected bladder and kidney of mice in the challenge assay. Two weeks after the last immunization, the bladders of mice were inoculated with 10^8^ cfu of UP*EC*. Colony count distribution of UP*EC* in the (a) bladder and (b) kidneys. Statistical analysis shows significant differences in the tFimH.FliC and FimH groups over the FliC group (*p* < 0.05). In addition, the significant difference of the tFimH.FliC and FimH with the control group was seen (*p* < 0.05).

**Figure:**
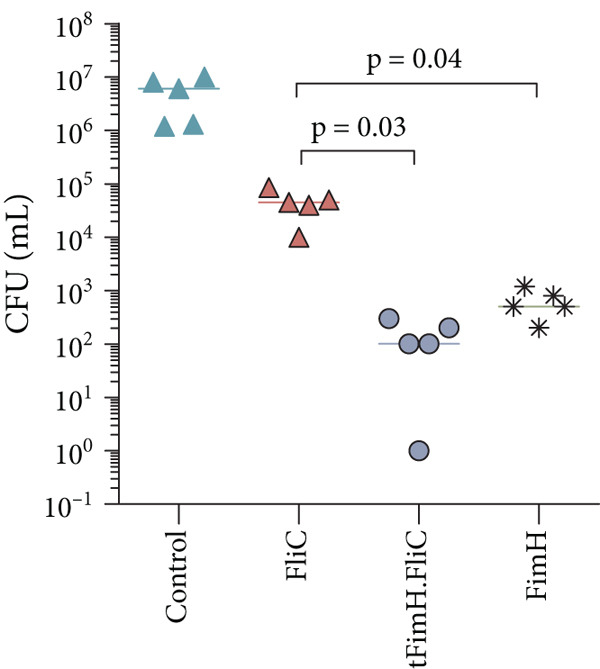
(a)

**Figure:**
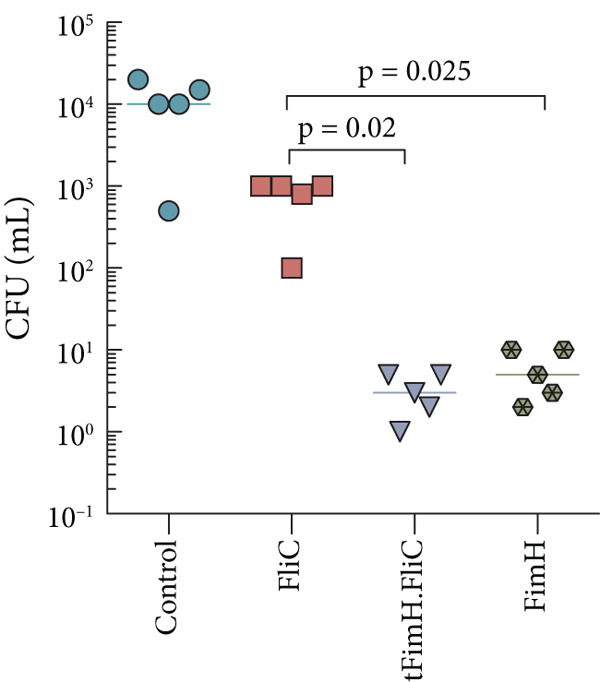
(b)

## 4. Discussion

Over the past two decades, there has been significant progress in developing a vaccine against UTIs. However, it is surprising that no licensed UTI vaccines are currently available for use in the world. Additionally, due to the increasing number of UP*EC* strains producing beta‐lactamases with a wide spectrum and the potential for resistance to carbapenem, there is an urgent need to develop a vaccine [28]. Therefore, it is necessary to evaluate different antigens, adjuvants, immunogenicity pathways, and novel technologies to achieve an effective vaccine against UTIs. Currently, the advancement of recombinant protein vaccines is hailed as a promising strategy to stimulate significant immune responses. Recombinant proteins are robust contenders for immunization against bacterial diseases because of their practical benefits of stability, safety, and high immunogenicity when used with adjuvants. Furthermore, they have shown promising results in clinical development [29].

Fusion of immunogenic proteins with appropriate adjuvants has been shown to enhance the immunogenicity of vaccines. Asadi Karam et al. have shown that fusion protein FimH.FliC was significantly more able to induce the immune system and protect mice against UP*EC* infection than the mixed preparation of the antigens [21]. The main reason for the significant immune system responses induced by the flagellin fusion protein is the better antigen processing and presentation, due to the presence of TLR‐5 ligands on the APCs [16, 30]. Also, several recent studies have shown that FimH interacts with TLR‐4 and thus can activate the immune system as an adjuvant [21, 31, 32]. In the present study, we designed and evaluated a fusion protein composed of a truncated form of FimH and FliC from *Salmonella typhimurium*. Of course, we constructed and used the best fusion protein model using bioinformatics analyses. The main antigens used in the vaccine candidates should be widely distributed among different clinical strains and possess epitopes that are conserved among the strains. Therefore, we used the UP*EC* CFT073 strain, whose sequence is identical to more than 90% of the sequences of other strains registered in NCBI. Also, unlike the complete form of FimH, a truncated form of FimH can overcome the expression problems of high molecular weight fusion proteins and eliminate the involuntarily domains that eventually will increase the vaccine′s effectiveness.

Evaluation of antibody responses showed that the fusion protein tFimH.FliC induced humoral and mucosal immune responses. As shown in Figure 5, the tFimH.FliC induced significant total IgG serum antibody, as well as both IgG1 (Th2) and IgG2a (Th1) isotypes compared to the control group. Although the FliC in the fusion protein could specifically increase both IgG1 and IgG2a antibody levels, the IgG1 response was higher than the IgG2a in the vaccinated mice. In the present study, the ratio of IgG1/IgG2a in tFimH.FliC and FimH groups was 1.63 and 2.12, respectively. This result suggests that the addition of FliC to tFimH in the fusion protein tFimH.FliC tended to switch the humoral responses toward Th1 or cellular response. In contrast to our findings, Honko et al. showed that FliC, with increasing the IgG1/IgG2a ratio, tended to bias the immune responses toward Th2 (antibody response) [33].

Although, in our study, the route of administration was subcutaneous, the humoral response was also stimulated in the mucosa through the significant production of IgA in the urine samples of tFimH.FliC group compared to the control (*p* < 0.05), which can be an advantage in neutralizing the uropathogens (Figure 6). In another study, Wieser et al. [15] reported that the use of a multiepitope vaccine candidate from the subcutaneous route had the potential to induce IgA antibody production. Inducing the IgG isotypes and IgA antibodies in this study showed that fusing FliC to the truncated FimH protein has a noticeable effect in strengthening the power of tFimH to induce the humoral and mucosal immune system.

Cytokine assay results showed that the injection of fusion protein tFimH.FliC resulted in the production of significantly higher levels of IFN‐*γ*, IL‐4, and IL‐17 than the control group (Figures 7a, 7b, and 7c). It should be noted that despite the increased production of IFN‐*γ* and IL‐4 cytokines in the tFimH.FliC and tFimH groups compared to the control, the output of IFN‐*γ* cytokine significantly increased in the tFimH.FliC group compared to tFimH (*p* < 0.05), which confirms that FliC has shifted the immune response more toward cellular immunity (Th1). Whether FliC drives mainly immune responses to Th1 or Th2 is unclear. Gupta et al. [34] reported that the FliC as an adjuvant shifted the immune response toward Th1 responses, while Nempont et al. [35] reported that FliC leads the responses toward Th2. Furthermore, Knudsen et al. indicated an increase in both Th1 and Th2 responses [36]. Similarly, the present study and our previous study showed that FliC as an adjuvant in fusion protein could significantly increase both IFN‐*γ* and IL‐4 as patterns indicative of Th1 and Th2 responses, respectively [25]. In fact, in the current study, tFimH.FliC significantly increased IgG1, IgG2a, IL‐4, and IFN‐*γ* levels. These results confirmed that FliC has increased both Th1 and Th2 responses. Since one of the problems of UTIs is the presence of intracellular bacterial communities (IBCs) and one of the reasons for the formation of recurrent UTIs, cellular immunity is required to eliminate these bacterial forms [37]. So, improving IFN‐*γ* and IL‐4 production levels confirmed the activation of two immune response pathways against UP*EC* that can be beneficial to eliminate the extracellular and intracellular uropathogenic sources. Also, FliC is used as an adjuvant to promote immunity responses and is applied as a TLR‐5 ligand to protect the urinary tract against pathogens by activating the TLR‐5 signaling pathway, based on results from many studies. Besides, using natural adjuvants such as FliC reduces the side effects for the host, as a result of using the commercial and synthetic adjuvants [38–40].

Our findings in the challenge test showed that mice vaccinated with tFimH.FliC and tFimH significantly reduced UP*EC* colonization in the bladder and kidney compared to the FliC and control groups (*p* < 0.05) (Figure 8). This protection among the vaccinated mice could be attributed to the cellular or humoral responses or a combination of both [41, 42]. As shown in Figure 8, the mice vaccinated with FimH and FliC alone tended to reduce the UP*EC* in the bladder and kidneys, confirming the important roles of FimH and FliC in the bladder colonization. The reduction of infections in the kidneys may be due to the role of FimH and FliC in the ascension of bacteria from the bladder to the kidneys or may be a direct relation between the reduction of UP*EC* in the bladder and the reduction of infection in the kidneys [23, 43, 44]. Interestingly, in the challenge test, the UP*EC* also decreased in the group vaccinated with the FliC of *Salmonella typhimurium*. Although this decrease was less than in other groups, it was unpredictable.

## 5. Conclusion

The results of this study showed that the tFimH.FliC fusion protein could induce significant immune responses and reduce the colonization of bacteria in the bladders and kidneys of mice. These findings show that protective immune responses can be created with the truncated form of the FimH protein, which is more economical for production and does not contain unwanted and nonimmunogenic domains. Although the tFimH.FliC fusion protein needs further investigation, it can be proposed as a vaccine candidate to protect the urinary tract against UP*EC* strains.

## Conflicts of Interest

The authors declare no conflicts of interest.

## Author Contributions

**Aslam Dehvari:** writing – original draft, methodology. **Mohammad Reza Asadi Karam:** writing – review and editing, validation, supervision, methodology, conceptualization. **Ebrahim Kord:** writing – original draft, writing – review and editing. **Shahram Shahraki Zahedani:** validation, supervision, conceptualization. **Mehri Habibi:** methodology, data curation, visualization. **Zabihollah Hashemzahi:** formal analysis, data curation. **Zakaria Bameri:** writing – original draft, writing – review and editing, methodology, visualization, formal analysis, data curation.

## Funding

This work was supported by Zahedan University of Medical Sciences (10.13039/501100004847, IR.ZAUMS.REC.1398.054).

## Data Availability

The data that support the findings of this study are available from the corresponding authors upon reasonable request.
